# Neuro-adaptive augmented distributed nonlinear dynamic inversion for consensus of nonlinear agents with unknown external disturbance

**DOI:** 10.1038/s41598-022-05663-4

**Published:** 2022-02-07

**Authors:** Sabyasachi Mondal, Antonios Tsourdos

**Affiliations:** Aerospace Engineering, Cranfield University, MK430AL, UK

**Keywords:** Engineering, Aerospace engineering, Electrical and electronic engineering

## Abstract

This paper presents a novel neuro-adaptive augmented distributed nonlinear dynamic inversion (N-DNDI) controller for consensus of nonlinear multi-agent systems in the presence of unknown external disturbance. N-DNDI is a blending of neural network and distributed nonlinear dynamic inversion (DNDI), a new consensus control technique that inherits the features of Nonlinear Dynamic Inversion (NDI) and is capable of handling the unknown external disturbance. The implementation of NDI based consensus control along with neural networks is unique in the context of multi-agent consensus. The mathematical details provided in this paper show the solid theoretical base, and simulation results prove the effectiveness of the proposed scheme.

## Introduction

Cooperation among agents, i.e., the consensus, is a fundamental and essential requirement to execute a complex task cooperatively. In a real-world scenario, the agents face a variety of issues while making the consensus. These issues are associated with communication among the agents, plant’s uncertainty and unknown external disturbances. The former does not affect the agent dynamics, but the latter does a lot resulting in a mission failure. Considering the importance of a mission, the researchers focused on designing adaptive controllers capable of handling unknown disturbances. These controllers implement adaptive control laws, including the neural network (NN) based approximation scheme and the conventional linear or nonlinear control theory depending on the plant dynamics. The primary reason for selecting the NN is that it is an efficient technique to approximate unknown nonlinear functions^1^, especially the radial basis function (RBF) neural network, which is widely used due to its simple structure. Such neuro-adaptive controllers are proposed to solve a variety of consensus problems. A few examples are mentioned here. A leader-follower synchronization problem for uncertain dynamical nonlinear agents was solved using neuro-adaptive scheme^2^. A cooperative tracking problem of agents with unknown dynamics^3^ was proposed using a neural network-based controller. A bipartite consensus^4^ was achieved using a neural network to learn the uncertainties of agents. Another leader-follower output consensus problem was solved^5^ using a neuro-adaptive controller for a class of uncertain heterogeneous non-affine pure-feedback multi-agent systems in the presence of time-delay and input saturation. An adaptive leader-following consensus control for a class of strict-feedback agents^6^ was solved using neuro-adaptive control. An exciting example of distributed finite-time formation tracking control problem for multiple unmanned helicopters was presented by Wang et al.^7^. The authors used the radial basis function neural network (RBFNN) technique to design a novel finite-time multivariable neural network disturbance observer (FMNNDO) to approximate the unknown external disturbance and model uncertainty law. In addition to nonlinear systems, a neural-network-based leaderless consensus control problem of fractional-order multi-agent systems (FOMASs) with unknown nonlinearities and unknown external disturbances was reported^8^. The effect of actuator fault on consensus asymptotic convergence of nonlinear agents with unknown dynamics was discussed by Li et al.^9^. Other examples include event-triggered consensus control problem for nonstrict-feedback nonlinear systems with a dynamic leader^10^, fixed-time leader-follower consensus problem for multi-agent systems (MASs) with output constraints, unknown control direction, unknown system dynamics, an unknown external disturbance^11^, stochastic nonlinear multi-agent systems with input saturation^12^ etc.

These papers implemented a variety of nonlinear controllers (e.g. feedback linearization, Lyapunov function, sliding mode, backstepping etc.) and a neural network approximation for uncertainty and unknown disturbances. In this paper, we have presented a neuro-adaptive augmented distributed controller, which is designed based on Distributed Nonlinear Dynamic Inversion (DNDI)^13^. We named it N-Distributed NDI (N-DNDI). It can be mentioned that the adaptive control expression in the papers mentioned earlier contains a linear or nonlinear error feedback term, and an adaptive term is added to it. However, N-DNDI is a new neuro-adaptive structure augmented in the DNDI frame. The primary reasons for selecting NDI are given as follows.

- The NDI is an effective way to design a controller for plants with nonlinear dynamics. The nonlinearities in the plant are eliminated by using feedback linearization theory. Moreover, the response of the closed-loop plant is similar to a stable linear system.
- The NDI controller has many advantages. Examples of these advantages include 1. simple and closed-form control expression, 2. easily implementable, global exponential stability of the tracking error, 3. use of nonlinear kinematics in the plant inversion, 4. minimize the need for individual gain tuning, etc.

Many researchers have used NDI to solve their research problems. Enns et al.^14^ implemented NDI to design a flight controller. Singh et al.^15^ developed a controller for autonomous landing of a UAV. Padhi et al.^16^ described reactive obstacle avoidance schemes for UAVs in a Partial Integrated Guidance and Control (PIGC) framework using neuro-adaptive augmented dynamic inversion. Mondal et al.^17^ applied NDI to propose a formation flying scheme. They presented how the NDI is implemented for tracking the leader’s commands in terms of coordinate, velocity, and orientation. Caverly et al.^18^ used NDI to control the attitude of a flexible aircraft. Horn et al.^19^ designed a controller of rotorcraft using Dynamic Inversion. Lombaerts et al.^20^ proposed NDI-based attitude control of a hovering quad tilt-rotor eVTOL Vehicle.

The contribution is given as follows.

- In this paper, a novel neuro-adaptive Distributed NDI (N-DNDI) is proposed to achieve the consensus among a class of nonlinear agents in the presence of unknown external disturbance. It can be mentioned that DNDI is a new consensus protocol^13^ and augmentation of the neural network with DNDI is a new formulation. Hence, this is new in the context of MASs and not reported in the literature.
- The main advantage of N-DNDI is it inherits the features of NDI. Moreover, the augmentation of the neural network provides a very good approximation of the unknown external disturbances. Therefore, N-DNDI is a perfect combination for designing consensus controllers for nonlinear agents. The realistic simulation study justifies the effectiveness of blending DNDI and neural networks.
- The formulation to accommodate the neuro-adaptive structure in the DNDI framework is a significant contribution. Moreover, the mathematical details for convergence are provided to show the solid theoretical base of this new controller.

The rest of the paper is organized as follows. In section “Preliminaries”, preliminaries are given. Section “Problem formulation” presents the problem definition. The mathematical details of the DNDI are provided in section “Nominal distributed nonlinear dynamic inversion (DNDI) controller”. The mathematical details of N-DNDI are given in section “Neuro-adaptive augmented DNDI for consensus”. The simulation study is presented in section “Simulation results”. The conclusion is given in section “Conclusion”.

## Preliminaries

The topics which are relevant to the problem considered in this paper are given in this section.

### Consensus of multiple agents

The consensus of MASs on communication network is discussed in this section. The definition of the consensus is given as follows.

#### Definition 1

Let us consider a MASs with *N* agents, where $X_i,\ (i = 1, 2, 3, . . . ,N)$ denotes the states of the *i*th agent. The MASs will achieve the consensus if $\parallel X_i - X_j \parallel \rightarrow 0, \forall i \ne j$ as $t\rightarrow +\infty$.

The consensus protocol aims to minimize the error in similar states of the individual agent with their neighbour by sharing information over the communication network, which is generally described using graph theory.

### Graph theory

The communication among the agents can be represented by a weighted graph written by $G= \{V, E\}$. The vertices $V=\{v_1, v_2,\ldots , v_N \}$ of the graph denote the agents, and the set of edges, denoted by $E\subseteq V\times V$, represents the communication among the agents. The weighted adjacency matrix $A=[a_{ij}]\in {\mathfrak {R}}^{N \times N}$ of *G* is denoted by $a_{ij}>0$ if $(v_j,v_i)\in E$, otherwise $a_{ij}=0$. There is no self loop in the graph. This fact is expressed by selecting the diagonal elements of the adjacency matrix *A* as zero, i.e., $i\in V$, $a_{ii}=0$. The degree matrix is denoted by $D \in {\mathfrak {R}}^{N\times N}=diag\{d_1\ d_2\ \ldots d_N\}$, where $d_i=\sum _{j\in N_i}a_{ij}$. The Laplacian matrix is written as $L=D-A$. A graph with the property that $a_{ij}=a_{ji}$ is said to be undirected graph. If any two nodes $v_i, v_j\in V$, there exists a path from $v_i$ to $v_j$, then the graph is called a connected graph. In this paper, we suppose that the topology *G* of the network is undirected and connected.

### Radial basis function neural networks (RBFNNs)

Due to the ‘linear in the weight’ property, the Neural networks are widely implemented to approximate unknown functions and the radial basis function neural network (RBFNN) is a good candidate^21^. A continuous unknown nonlinear function $\zeta (X):{\mathfrak {R}}^n\rightarrow {\mathfrak {R}}^m$ can be approximated by

1 $$\begin{aligned} \zeta (X)= W_{NN}^T \Phi (X) + \epsilon _X \end{aligned}$$

where $X\in {\mathfrak {R}}^n$ is input vector, $W_{NN}\in {\mathfrak {R}}^{q \times m}$ is the weights of RBFs, $\Phi (X)=[\phi _1(X)\ \ldots \ \phi _q(X) ]^T$ denotes the basis function vector. ‘*q*’ denotes the number of neurons. $\epsilon _X\in {\mathfrak {R}}^m$ is the approximation error. The *i*th basis function $\phi _i$ is given by

2 $$\begin{aligned} \phi _i(X)=\exp ^{\frac{(X-\mu _i)^T(X-\mu _i) }{\psi _i^2}}\quad ;i=1,2, \ldots , q. \end{aligned}$$

where $\mu _i\in {\mathfrak {R}}^n$ is the center of the receptors and $\psi _i$ is width of the *i*th gaussian function.

### Useful lemma

The useful lemmas used in this paper are given as follows.

#### Lemma 1

^22^ *The Laplacian matrix* *L* *in an undirected graph is semi-positive definite, it has a simple zero eigenvalue and all the other eigenvalues are positive if and only if the graph is connected. Therefore*, *L* *is symmetric and it has* *N* *non-negative, real-valued eigenvalues* $0=\lambda _1\le \lambda _2\le \ldots \le \lambda _N$.

#### Lemma 2

^23^ *Let* $\psi _1(t), \psi _2(t)\in R^m$ *be continuous positive vector functions, by Cauchy inequality and Young’s inequality, there exists the following inequality*:

3 $$\begin{aligned} \psi _1(t)\psi _2(t) &\le \parallel \psi _1(t) \parallel \parallel \psi _2(t) \parallel \\ &\le \frac{\parallel \psi _1(t) \parallel ^\lambda }{\lambda }+\frac{\parallel \psi _2(t) \parallel ^\zeta }{\zeta } \end{aligned}$$

*where*

$$\begin{aligned} \frac{1}{\lambda }+\frac{1}{\zeta }=1 \end{aligned}$$

#### Lemma 3

^24^ *Let* $R(t)\in {\mathfrak {R}}$ *be a continuous positive function with bounded initial* *R*(0). *If the inequality holds* ${\dot{R}}(t) \le -\beta R(t)+\eta$ *where*, $\beta>0, \eta >0$, *then the following inequality holds.*

4 $$\begin{aligned} R(t) \le R(0) e^{-\beta t}+\frac{\eta }{\beta }\left( 1-e^{-\beta t} \right) \end{aligned}$$

## Problem formulation

In this section, the problem definition is given. The objective is to design a neuro-adaptive consensus protocol that enables a class of nonlinear agents to achieve the consensus in the presence of external disturbance. Let us consider a group of *N* nonlinear agents. They are connected by the undirected and connected network topology. All the agents are homogeneous, i.e., they have similar dynamics. The dynamics of *i*th agent is given by Eqs. (5)–(6) as follows.

5 $$\begin{aligned} \dot{X_i}= f(X_i)+g(X_i)U_i+D_i(X_i) \end{aligned}$$

6 $$\begin{aligned} Y_i= X_i \end{aligned}$$

where, $X_i\in {\mathfrak {R}}^n$, $U_i\in {\mathfrak {R}}^n$ are states and control respectively. *f* is a continuously differentiable vector-valued function representing the nonlinear dynamics. $D_i(X_i)\in {\mathfrak {R}}^n$ is the unknown bounded and smooth external disturbance term with $\forall t\ge 0$.

### Assumption 1

The matrix $g(X_i)$ is invertible for all time.

## Nominal distributed nonlinear dynamic inversion (DNDI) controller

It is relevant to get an overview of the DNDI controller^13^ and its convergence behaviour before augmenting neuro-adaptive structure is explained.

### Brief overview of DNDI

**Figure 1:**
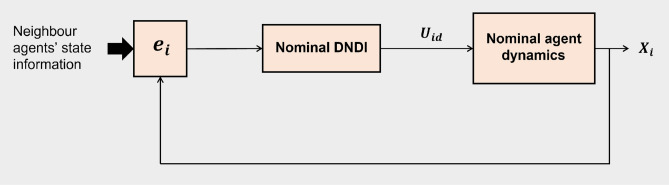
Block diagram of distributed NDI or DNDI.

A brief overview of DNDI controller is presented here. The block diagram of the consensus control scheme with nominal DNDI is shown in the Fig. 1.

The nominal dynamics of *i*th agent is given as follows.

7 $$\begin{aligned} \dot{X_i}= f(X_i)+g(X_i)U_{id} \end{aligned}$$

8 $$\begin{aligned} Y_i= X_i \end{aligned}$$

where, $X_i\in {\mathfrak {R}}^n$, $U_{id}\in {\mathfrak {R}}^n$ . ${\mathbf {e}}_i$ denotes the consensus error of *i*th agent given by

9 $$\begin{aligned} {\mathbf {e}}_i = {\bar{d}}_i X_i-{\bar{a}}_i {\mathbf {X}} \end{aligned}$$

where ${\mathbf {e}}_i\in {\mathfrak {R}}^n$, ${\bar{d}}_i=(d_i \otimes {\mathbf {I}}_n) \in {\mathfrak {R}}^{n \times n}$, ${\bar{a}}_i=(a_i \otimes {\mathbf {I}}_n) \in {\mathfrak {R}}^{n \times nN}$, and ${\mathbf {X}}=[X^T_1\ X^T_2\ \ldots \ X^T_N]^T \in {\mathfrak {R}}^{nN}$. ${\mathbf {I}}_n$ is $n\times n$ identity matrix. ‘$\otimes$’ denotes the Kroneker product. Enforcing the first order error dynamics we get

10 $$\begin{aligned} \dot{{\mathbf {e}}}_i+K_i {\mathbf {e}}_i=0 \end{aligned}$$

Differentiation of Eq. (9) yields

11 $$\begin{aligned} \dot{{\mathbf {e}}}_i&= {\bar{d}}_i {\dot{X}}_i - {\bar{a}}_i \dot{{\mathbf {X}}} \\&= {\bar{d}}_i \left( f(X_i)+ g(X_i)U_{id} \right) -{\bar{a}}_i \dot{{\mathbf {X}}} \end{aligned}$$

Substitution of the expressions for ${\mathbf {e}}_i$ and $\dot{{\mathbf {e}}}_i$ in Eq. (10) gives

12 $$\begin{aligned} {\bar{d}}_i \left( f(X_i)+ g(X_i)U_{id}\right) -{\bar{a}} \dot{{\mathbf {X}}} + K_i ({\bar{d}}_i X_i-{\bar{a}}_i {\mathbf {X}})=0 \end{aligned}$$

Simplification of Eq. (12) gives the expression of control $U_{id}$ for *i*th agent as follows.

13 $$\begin{aligned} U_{id} = (g(X_i))^{-1} \left[ -f(X_i)+{\bar{d}}^{-1}_i({\bar{a}}_i \dot{{\mathbf {X}}}-K_i ({\bar{d}}_i X_i - {\bar{a}}_i {\mathbf {X}}))\right] \end{aligned}$$

### Convergence of DNDI

Convergence study of DNDI is presented here. Let us consider a smooth scalar function given by

14 $$\begin{aligned} V=\frac{1}{2} {\mathbf {X}}^T(L\otimes {\mathbf {I}}_n) {\mathbf {X}} \end{aligned}$$

$L\otimes {\mathbf {I}}_n$ can be written as

15 $$\begin{aligned} L\otimes {\mathbf {I}}_n=S\Delta S^T \end{aligned}$$

where, $S\in {\mathfrak {R}}^{nN\times nN}$ is the left eigenvalue matrix of $L\otimes {\mathbf {I}}_n$, $\Delta =\left( diag\{ 0,\lambda _2(L), \lambda _3(L), \ldots , \lambda _N(L)\}\otimes {\mathbf {I}}_n \right) \in {\mathfrak {R}}^{nN\times nN}$ is eigenvalue matrix, $S^TS=SS^T={\mathbf {I}}_{nN\times nN}$.

16 $$\begin{aligned} V&= \frac{1}{2}{\mathbf {X}}^T(L\otimes {\mathbf{I }}_n){\mathbf {X}} \\&= \frac{1}{2}{\mathbf {X}}^TS\Delta S^T {\mathbf {X}} \\&= \frac{1}{2}{\mathbf {X}}^TS \sqrt{\Delta } \sqrt{\Delta } S^T{\mathbf {X}} \\&= \frac{1}{2}{\mathbf {X}}^T S \sqrt{\Delta {\bar{\Delta }}}\sqrt{{\bar{\Delta }}^{-1}} \sqrt{{\bar{\Delta }}^{-1}} \sqrt{{\bar{\Delta }} \Delta } S^T {\mathbf {X}} \\&= \frac{1}{2}{\mathbf {X}}^T S \Delta {\bar{\Delta }}^{-1} \Delta S^T{\mathbf {X}} \\&= \frac{1}{2}{\mathbf {X}}^T S \Delta \left( S^T S\right) {\bar{\Delta }}^{-1} \left( S^T S \right) \Delta S^T{\mathbf {X}} \\&= \frac{1}{2}{\mathbf {X}}^T \left( S \Delta S^T\right) \left( S {\bar{\Delta }}^{-1} S^T\right) \left( S \Delta S^T\right) {\mathbf {X}} \\&= \frac{1}{2}{\mathbf {X}}^T(L\otimes {\mathbf {I}}_n)\Lambda (L\otimes {\mathbf{I }}_n){\mathbf {X}} \\&= \frac{1}{2}E^T\Lambda E \end{aligned}$$

where $${\bar{\Delta }}=\left( diag\{\lambda _2(L),\lambda _2(L), \lambda _3(L), \ldots , \lambda _N(L)\}\otimes {\mathbf {I}}_n \right) \in {\mathfrak {R}}^{nN\times nN}$$, $$E=[{\mathbf{e }}^T_1\ {\mathbf{e }}^T_2\ \ldots \ {\mathbf{e }}^T_N]^T\in {\mathfrak {R}}^{nN}$$, and $$\Lambda =S {\bar{\Delta }}^{-1} S^T \in {\mathfrak {R}}^{nN \times nN}$$.

#### Remark 1

It can be observed from Eqs. (14) and (16) that

17 $$\begin{aligned}\frac{\lambda _{min}(\Lambda )}{2} \parallel E \parallel ^2 \le V \le \frac{\lambda _{max}(\Lambda )}{2} \parallel E \parallel ^2 \end{aligned}$$

18 $$\begin{aligned}V=\frac{1}{2}{\mathbf {X}}^T(L\otimes {\mathbf {I}}_n){\mathbf {X}}=\frac{1}{2}{\mathbf {X}}^TE \end{aligned}$$

#### Remark 2

According to Lemma 1, $\lambda _2>0$. Hence, ${\bar{\Delta }}$ is invertible.

#### Remark 3

$\Lambda =S {\bar{\Delta }}^{-1} S^T$ is positive definite matrix. Hence, *V* is positive definite subject to consensus error and qualify for a Lyapunov function.

Differentiating Eq. (14), we get

19 $$\begin{aligned} {\dot{V}}={\mathbf {X}}^T(L\otimes {\mathbf {I}}_n) \dot{{\mathbf {X}}}=E^T \dot{{\mathbf {X}}}=\sum ^N_{i=1}{\mathbf {e}}^T_i\left[ f(X_i)+g(X_i)U_{id}\right] \end{aligned}$$

where, $E=[{\mathbf {e}}^T_1\ {\mathbf {e}}^T_2\ \ldots \ {\mathbf {e}}^T_N]^T\in {\mathfrak {R}}^{nN}$. Substituting the control $U_{id}$ expression in Eq. (19) yields

20 $$\begin{aligned} {\dot{V}}&= \sum ^N_{i=1}{\mathbf {e}}^T_i\left[ {\bar{d}}^{-1}_i({\bar{a}}_i \dot{\mathbf{X }}-K_i {\mathbf{e }}_i)\right] \\&= \sum ^N_{i=1}-{\mathbf {e}}^T_i{\bar{d}}^{-1}_iK_i {\mathbf {e}}_i+\sum ^N_{i=1}{\mathbf {e}}^T_i{\bar{d}}^{-1}_i{\bar{a}}_i \dot{{\mathbf {X}}} \end{aligned}$$

According to Lemma 2, we can write

21 $$\begin{aligned} {\mathbf {e}}^T_i{\bar{d}}^{-1}_i{\bar{a}}_i \dot{{\mathbf {X}}}\le \parallel {\mathbf {e}}_i \parallel \ \parallel {\bar{d}}^{-1}_i{\bar{a}}_i \dot{{\mathbf {X}}} \parallel \le \frac{\parallel {\mathbf {e}}_i \parallel ^2}{2}+\frac{\parallel {\bar{d}}^{-1}_i{\bar{a}}_i \dot{{\mathbf {X}}} \parallel ^2}{2} \end{aligned}$$

Substituting the inequality relation in Eq. (20)

22 $$\begin{aligned} {\dot{V}} \le \sum ^N_{i=1}\left[ -{\mathbf {e}}^T_i{\bar{d}}^{-1}_iK_i {\mathbf {e}}_i+\frac{\parallel {\mathbf {e}}_i \parallel ^2}{2}+\frac{\parallel {\bar{d}}^{-1}_i{\bar{a}}_i \dot{{\mathbf {X}}} \parallel ^2}{2}\right] \end{aligned}$$

Let us design the gain $K_i$ as follows.

23 $$\begin{aligned} K_i={\bar{d}}_i\left( \frac{1}{2}+\frac{\alpha _i}{2}\lambda _{max}(\Lambda ) \right) \end{aligned}$$

Eq. (22) is written as

24 $$\begin{aligned} {\dot{V}}&\le \sum ^N_{i=1}\left[ - \frac{\alpha _i}{2}\lambda _{max}(\Lambda ) \parallel {\mathbf {e}}_i \parallel ^2+\frac{\parallel {\bar{d}}^{-1}_i{\bar{a}}_i \dot{{\mathbf {X}}} \parallel ^2}{2} \right] \\ &\le -\alpha _i V+ \eta \end{aligned}$$

where, $\eta =\sum ^N_{i=1} \frac{\parallel {\bar{d}}^{-1}_i{\bar{a}}_i \dot{{\mathbf {X}}} \parallel ^2}{2}$. Applying Lemma 3 we get

25 $$\begin{aligned} V \le \frac{\eta }{\alpha _i}+\left( V(0)-\frac{\eta }{\alpha _i} \right) e^{-\alpha _i t} \end{aligned}$$

Hence, we conclude that *V* is bounded as $t\rightarrow \infty$. In addition, we show the Uniformly Ultimate Boundedness (UUB) here.

Using Eq. (17), Eq. (25), and $Lemma\ 1.2$ presented by Ge et al.^24^ we can write

26 $$\begin{aligned} \frac{\lambda _{min}(\Lambda )}{2} \parallel E \parallel ^2 \le V \le \frac{\eta }{\alpha _i}+\left( V(0)-\frac{\eta }{\alpha _i} \right) e^{-\alpha _i t} \end{aligned}$$

Eq. (26) can be written as follows.

27 $$\begin{aligned} \frac{\lambda _{min}(\Lambda )}{2} \parallel E \parallel ^2 &\le \frac{\eta }{\alpha _i}+\left( V(0)-\frac{\eta }{\alpha _i} \right) e^{-\alpha _i t} \\ \parallel E \parallel &\le \sqrt{\frac{2\frac{\eta }{\alpha _i}+2\left( V(0)-\frac{\eta }{\alpha _i} \right) e^{-\alpha _i t}}{\lambda _{min}(\Lambda )}} \end{aligned}$$

It can be observed that, if $V(0)= \frac{\eta }{\alpha _i}$ then

28 $$\begin{aligned} \parallel E \parallel \le \kappa ^* \end{aligned}$$

$\forall t \ge 0$ and $\kappa ^* = \sqrt{\frac{2\eta }{\alpha _i\lambda _{min}(\Lambda )}}$. If $V(0)\ne \frac{\eta }{\alpha _i}$ then for any given $\kappa >\kappa ^*$ there exist a time $T>0$ such that $\forall t>T$, $\parallel E \parallel \le \kappa$.

29 $$\begin{aligned} \kappa = \sqrt{\frac{2\frac{\eta }{\alpha _i}+2\left( V(0)-\frac{\eta }{\alpha _i} \right) e^{-\alpha _i T}}{\lambda _{min}(\Lambda )}} \end{aligned}$$

Therefore, we can conclude

30 $$\begin{aligned} \lim _{t\rightarrow \infty } \parallel E \parallel = \kappa ^* \end{aligned}$$

## Neuro-adaptive augmented DNDI for consensus

Before going to the main derivation of Neuro-adaptive DNDI, we present the philosophy of neuro-adaptive control design^25^.

### Philosophy of neuro-adaptive control

**Figure 2:**
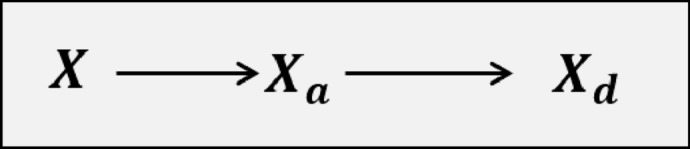
Philosophy of neuro-adaptive control.

The sole objective of the design is to drive the actual state *X* to desired state $X_d$. The scheme adopted is to make actual state *X* to track the desired or nominal state $X_d$ through the virtual state $X_a$ as shown in Fig. 2.

The tracking of *X* to $X_a$ and $X_a$ to $X_d$ is achieved by enforcing error dynamics to obtain the control considering nonlinear plant dynamics. We use the same philosophy to design the Neuro-adaptive distributed NDI controller in the next section.

### Mathematical details of neuro-adaptive augmented DNDI (N-DNDI)

**Figure 3:**
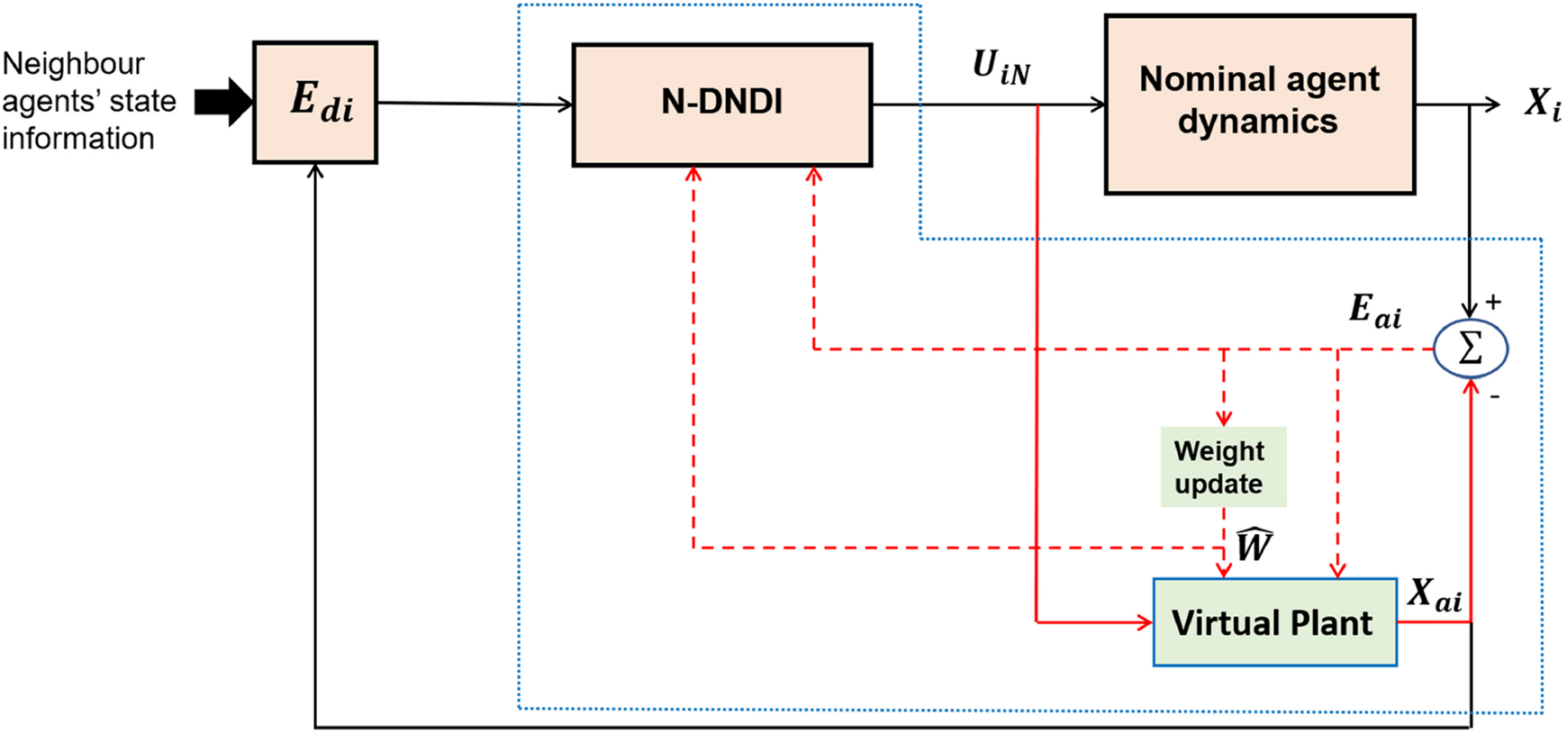
Block diagram of Neuro-adaptive DNDI or N-DNDI.

Neuro-adaptive augmented DNDI is a blending of neuro-adaptive control and DNDI. The block diagram of the control scheme is shown in Fig. 3. The portion of the diagram inside the blue border is the proposed design of neuro-adaptive controller.

In case of neuro-adaptive augmented DNDI, the consensus error of *i*th agent is defined such that, the virtual state of *i*th agent, i.e., $X_{ai}\in {\mathfrak {R}}^n$ reach consensus with the neighbours. Therefore, the consensus error of *i*th agent is given by

31 $$\begin{aligned} E_{di}&= \sum _{j \in N_i} a_{ij} (X_{ai} - X_j ) \\&= {\bar{d}}_i X_{ai} - {\bar{a}}_i {\mathbf {X}} \end{aligned}$$

where $E_{di}\in {\mathfrak {R}}^n$. ${\mathbf {X}}\in {\mathfrak {R}}^{nN}$ denotes the actual states of all the agents. The actual dynamics of *i*th agent is given by

32 $$\begin{aligned} {\dot{X}}_i=f(X_i)+g(X_i)U_{iN}+D(X_i) \end{aligned}$$

where $D(X_i)$ is the external disturbance added to *i*th agent. The virtual dynamics for *i*th agent is given by

33 $$\begin{aligned} {\dot{X}}_{ai}=f(X_i)+g(X_i) U_{iN}+ {\hat{D}}(X_i)+K_{ai}(X_i-X_{ai}) \end{aligned}$$

where ${\hat{D}}(X_i)$ is the approximation of $D(X_i)$.

It is important to note that, the consensus error $E_{di}$ in Eq. (31) is designed to measure the error in virtual state of *i*th agent and actual states of its neighbours. To drive this error to zero (i.e., $E_{di}\rightarrow 0$), we define a Lyapunov function $V_i$ as follows.

34 $$\begin{aligned} V_i=\frac{1}{2}{E}_{di}^T{E}_{di} \end{aligned}$$

Differentiating Eq. (34) yields

35 $$\begin{aligned} {\dot{V}}_i={E}_{di}^T \dot{{E}}_{di} \end{aligned}$$

According to the Lyapunov stability theory, let the time derivative of the Lyapunov function should be

36 $$\begin{aligned} {\dot{V}}_i=-{E}_{di}^TK_{di}{E}_{di} \end{aligned}$$

where $K_{di} \in {\mathfrak {R}}^{n\times n}$ is a positive definite diagonal matrix. The expression of ${\dot{V}}_i$ in Eqs. (35) and (36) are equated to obtain

37 $$\begin{aligned} {E}_{di}^T \dot{{E}}_{di}=-{E}_{di}^TK_{di}{E}_{di} \end{aligned}$$

Eq. (37) is simplified as follows

38 $$\begin{aligned} {\dot{E}}_{di}+K_{di} E_{di}=0 \end{aligned}$$

Substituting the expression of $E_{di}$ in Eq. (38) we obtain

39 $$\begin{aligned} {\bar{d}}_i {\dot{X}}_{ai} - {\bar{a}}_i \dot{{\mathbf {X}}}+K_{di} ({\bar{d}}_i X_{ai} - {\bar{a}}_i {\mathbf {X}})=0 \end{aligned}$$

Putting the expression of ${\dot{X}}_{ai}$ in Eq. (39) yields

40 $$\begin{aligned} {\bar{d}}_i \left( f(X_i)+g(X_i) U_{iN}+ {\hat{D}}(X_i)+K_{ai}(X_i-X_{ai})\right) -{\bar{a}}_i \dot{{\mathbf {X}}}+K_{di} ({\bar{d}_i} X_{ai} - {\bar{a}}_i {\mathbf {X}})=0 \end{aligned}$$

The expression of control $U_{iN}$ can be obtained by simplifying Eq. (40) as follows.

41 $$\begin{aligned} U_{iN}= [g(X_i)]^{-1} \big [-f(X_i)-{\hat{D}}(X_i)-K_{ai}(X_i-X_{ai})+ {\bar{d}}_i^{-1}\left( {\bar{a}}_i \dot{{\mathbf {X}}}-K_{di} ({\bar{d}}_i X_{ai} - {\bar{a}}_i {\mathbf {X}}) \right) \big ] \end{aligned}$$

It can be observed that the control expression in Eq. (41) is different from Eq. (13). Next, the error dynamics is enforced for driving the actual state of *i*th agent to its virtual state, i.e., $X_i \rightarrow X_{ai}$.

42 $$\begin{aligned} {\dot{E}}_{ai}+K_{ai} E_{ai}=D(X_i)-{\hat{D}}(X_i) \end{aligned}$$

where $E_{ai}=X_i-X_{ai}$. To approximate the unknown disturbance a single layer neural network is designed as shown in Eq. (43).

43 $$\begin{aligned} {\hat{D}}(X_i)={\hat{W}}_i^T\Phi (X_i) \end{aligned}$$

where $\Phi (X_i)$ is a basis function vector. It is important to note that the ideal value of ${\hat{W}}_i$ is $W_i$ and thus the disturbance $D(X_i)$ can be approximated by

44 $$\begin{aligned} D(X_i)=W_i^T\Phi (X_i)+\epsilon _{X_i} \end{aligned}$$

where $\epsilon _{X_i}$ is the error tolerance. Eq. (42) is rewritten as

45 $$\begin{aligned} {\dot{E}}_{ai}+K_{ai} E_{ai}={\tilde{W}}_i^T \Phi (X_i)+\epsilon _{X_i} \end{aligned}$$

where ${\tilde{W}}_i=W_i-{\hat{W}}_i$. The weight update rule is given by

46 $$\begin{aligned} \dot{{\hat{W}}}_i=\gamma _i\left[ \Phi (X_i)E_{ai}^T-\sigma _i {\hat{W}}_i \right] \end{aligned}$$

where $\gamma _i$ is learning rate and $\sigma _i$ is stabilizing factor of *i*th agent. It is important to note that $\dot{{\tilde{W}}}_i=-\dot{{\hat{W}}}_i$ because $W_i$ is constant and ${\dot{W}}_i=0$.

### Convergence study of $E_{ai}$

The convergence study of the error $E_{ai}$ is important. We have selected a Lyapunov function as follows.

47 $$\begin{aligned} V_i= \frac{1}{2}E_{ai}^T E_{ai}+ \frac{1}{2} {\tilde{W}}^T_i \left( \gamma _i^{-1}\right) {\tilde{W}}_i \end{aligned}$$

48 $$\begin{aligned}= V_{E_{ai}}+V_{{\tilde{W}}_i} \end{aligned}$$

where $$V_{E_{ai}}=\frac{1}{2}E_{ai}^T E_{ai}$$ and $$V_{{\tilde{W}}_i}=\frac{1}{2} {\tilde{W}}^T_i \left( \gamma _i^{-1}\right) {\tilde{W}}_i$$.

Differentiation of Eq. (48) yields

49 $$\begin{aligned} {\dot{V}}_i&= E_{ai}^T {\dot{E}}_{ai} + {\tilde{W}}^T_i \gamma _i^{-1} \dot{{\tilde{W}}}_i \\&= E_{ai}^T \left( {\dot{X}}_i-{\dot{X}}_{ai} \right) -{\tilde{W}}^T_i \gamma _i^{-1} \gamma _i\left[ \Phi (X_i)E_{ai}^T-\sigma _i {\hat{W}}_i \right] \\&= E_{ai}^T \left( {\tilde{W}}^T_i\Phi (X_i)+\epsilon _{X_i}-K_{ai}E_{ai} \right) - {\tilde{W}}^T_i\left[ \Phi (X_i)E_{ai}^T -\sigma _i {\hat{W}_i} \right] \\&= \left( E_{ai}^T \epsilon _{X_i}-E_{ai}^TK_{ai} E_{ai} \right) +\sigma _i {\tilde{W}}^T_i {\hat{W}}_i \end{aligned}$$

Using Lemma 2 and ${\hat{W}}_i=-{\tilde{W}}_i+W_i$, Eq. (49) is written as

50 $$\begin{aligned} {\dot{V}}_i &\le \frac{ \parallel E_{ai} \parallel ^2}{2}+\frac{ \parallel \epsilon _{X_i} \parallel ^2}{2}- E_{ai}^TK_{ai} E_{ai} -\sigma _i \parallel {\tilde{W}}_i \parallel ^2+ \sigma _i \parallel {\tilde{W}}_i \parallel \parallel {W}_i \parallel \\ &\le \frac{ \parallel E_{ai} \parallel ^2}{2}+\frac{ \parallel \epsilon _{X_i} \parallel ^2}{2} -E_{ai}^TK_{ai} E_{ai} -\sigma _i \parallel {\tilde{W}}_i \parallel ^2 +\frac{1}{2} \sigma _i \parallel {\tilde{W}}_i \parallel ^2 +\frac{1}{2} \sigma _i \parallel {W}_i \parallel ^2 \\&= \frac{ \parallel E_{ai} \parallel ^2}{2}+ \frac{ \parallel \epsilon _{X_i} \parallel ^2}{2} -E_{ai}^TK_{ai} E_{ai} -\frac{1}{2} \sigma _i \parallel {\tilde{W}}_i \parallel ^2 +\frac{1}{2} \sigma _i \parallel {W}_i \parallel ^2 \\&= \frac{ \parallel E_{ai} \parallel ^2}{2} -E_{ai}^TK_{ai} E_{ai} -\frac{1}{2} \sigma _i \parallel {\tilde{W}}_i \parallel ^2 + \zeta _i \end{aligned}$$

where $\zeta _i=\frac{ \parallel \epsilon _{X_i} \parallel ^2}{2}+\frac{1}{2}\sigma _i \parallel {W}_i \parallel ^2$. Let us define

$$\begin{aligned} K_{ai}=\delta _i \left( \frac{1}{2\delta _i}+\frac{1}{2}\right) \quad \text {and} \quad \sigma _i \ge \delta _i \lambda _{max}(\gamma ^{-1}_i) \end{aligned}$$

where, $\delta _i>0$. Hence, we can write the Eq. (50) as follows.

51 $$\begin{aligned} {\dot{V}}_i\le -\frac{\delta _i}{2} \parallel E_{ai} \parallel ^2-\frac{\delta _i \lambda _{max}(\gamma ^{-1}_i)}{2} \parallel {\tilde{W}}_i \parallel ^2 + \zeta _i \end{aligned}$$

Using Eq. (17) we can write

52 $$\begin{aligned} \frac{1}{2} \parallel E_{ai} \parallel ^2\le V_{E_{ai}} \le V_i \end{aligned}$$

53 $$\begin{aligned} \frac{\lambda _{min}(\gamma ^{-1}_i)}{2} \parallel {\tilde{W}}_i \parallel ^2\le V_{{\tilde{W}}_i}\le V_i \end{aligned}$$

Therefore, Eq. (51) is written as follows.

54 $$\begin{aligned} {\dot{V}}_i\le -\delta _i V_{E_{ai}}-\delta _i V_{{\tilde{W}}_i}+\zeta _i \end{aligned}$$

55 $$\begin{aligned}= -\delta _i V_i+\zeta _i \end{aligned}$$

Applying Lemma 3 we can write

56 $$\begin{aligned} V_i(t) \le \frac{\zeta _i}{\delta _i}+\left( V_i(0)-\frac{\zeta _i}{\delta _i} \right) e^{-\delta _i t} \end{aligned}$$

#### Lemma 4

^24^ *Consider the positive function given by*

57 $$\begin{aligned} V =\frac{1}{2}e(t)^TQ(t)e(t)+ \frac{1}{2} {\tilde{W}}^T_i \left( \Gamma _i^{-1}\right) {\tilde{W}}_i \end{aligned}$$

*where* $e(t)=x(t)-x_d(t)$ and ${\tilde{W}}={\hat{W}}-W^*$. *If the following inequality holds*:

58 $$\begin{aligned} {\dot{V}}(t) \le -c_1V(t)+c_2 \end{aligned}$$

*then, given any initial compact set defined by*

59 $$\begin{aligned} \Omega _0=\left\{ x(0),x_d(0),{\hat{W}}(0) \vert x(0), {\hat{W}}(0)finite, x_d(0) \in \Omega _d \right\} \end{aligned}$$

*we can conclude that*

1. *the states and weights in the closed-loop system will remain in the compact set defined by*
  60 $$\begin{aligned} \Omega =\left\{ x(t),{\hat{W}}(t)\vert \parallel x(t) \parallel \le C_{e\ max}+ \max _{\tau \in [0,t]} \{\parallel x_d(\tau ) \parallel \}, x_d(t) \in \Omega _d, \parallel {\hat{W}} \parallel \le C_{{\tilde{W}}\ max}+\parallel W^* \parallel \right\} \end{aligned}$$
2. *the states and weights will eventually converge to the compact sets defined by*
  61 $$\begin{aligned} \Omega _s=\left\{ x(t),{\hat{W}}(t)\vert \lim _{t\rightarrow \infty } \parallel e(t) \parallel = \mu _e^*, \lim _{t\rightarrow \infty } \parallel {\tilde{W}}(t) \parallel = \mu _{{\tilde{W}}}^* \right\} \end{aligned}$$
  *where constants*
  62 $$\begin{aligned} C_{e\ max}= \sqrt{\frac{2V(0)+\frac{2c_2}{c_1}}{\lambda _{Q \ min}}} \end{aligned}$$
  63 $$\begin{aligned} C_{{\tilde{W}}\ max}= \sqrt{\frac{2V(0)+\frac{2c_2}{c_1}}{\lambda _{\Gamma \ min}}} \end{aligned}$$
  64 $$\begin{aligned} \mu _e^*= \sqrt{\frac{2c_2}{c_1 \lambda _{Q \ min}}} \end{aligned}$$
  65 $$\begin{aligned} \mu _{{\tilde{W}}}^*= \sqrt{\frac{2c_2}{c_1 \lambda _{\Gamma \ min}}} \end{aligned}$$

We will present the Uniformly Ultimate Boundedness (UUB) here using conclusion 2. Using Eqs. (52), (53), and (56) we can write

66 $$\begin{aligned} \parallel E_{ai} \parallel\le \sqrt{2\frac{\zeta _i}{\delta _i}+2\left( V_i(0)-\frac{\zeta _i}{\delta _i} \right) e^{-\delta _i t}} \end{aligned}$$

67 $$\begin{aligned} \parallel {\tilde{W}}_i \parallel\le \sqrt{\frac{2\frac{\zeta _i}{\delta _i}+2\left( V_i(0)-\frac{\zeta _i}{\delta _i} \right) e^{-\delta _i t}}{\lambda _{min}(\gamma ^{-1}_i)}} \end{aligned}$$

If $$V_i(0)=\frac{\zeta _i}{\delta _i}$$ then $$\parallel E_{ai} \parallel \le \mu ^*_{E_{ai}}$$, $$\forall t>0$$.

$$\begin{aligned} \mu ^*_{E_{ai}}=\sqrt{\frac{2\zeta _i}{\delta _i}} \end{aligned}$$

If $V_i(0)\ne \frac{\zeta _i}{\delta _i}$ then for a given $\mu _{E_{ai}}>\mu ^*_{E_{ai}}$ there exist a $T_E>0$ such that $\forall t>T_E$, we get $\parallel E_{ai} \parallel \le \mu _{E_{ai}}$

68 $$\begin{aligned} \mu _{E_{ai}} = \sqrt{2\frac{\zeta _i}{\delta _i}+2\left( V(0)-\frac{\zeta _i}{\delta _i} \right) e^{-\alpha _i T_E}} \end{aligned}$$

Therefore, we conclude

69 $$\begin{aligned} \lim _{t\rightarrow \infty }\parallel E_{ai} \parallel =\mu ^*_{E_{ai}} \end{aligned}$$

In a similar fashion, we can conclude

70 $$\begin{aligned} \lim _{t\rightarrow \infty }\parallel {\tilde{W}}_i \parallel =\mu ^*_{{\tilde{W}}_i} \end{aligned}$$

Therefore, according to conclusion 2, the proposed controller is able to make the approximation error to converge in the compact set defined by $\Omega _s$.

## Simulation results

Simulation results are presented here. The simulation study is performed on PC with AMD Ryzen 5 processor and 8 Gb RAM.

### Agent dynamics

The agent dynamics are given as follows.

71 $$\begin{aligned} {\dot{X}}_{i_1}= X_{i_2} \sin (2X_{i_1})+U_{i_1} \end{aligned}$$

72 $$\begin{aligned} {\dot{X}}_{i_2}= X_{i_1} \cos (3X_{i_2})+U_{i_2} \end{aligned}$$

where $X_i=\left[ X_{i_1} \ X_{i_2} \right] ^T$. Equations (71) and (72) give

73 $$\begin{aligned} f(X_i)=\begin{bmatrix} X_{i_2} \sin (2X_{i_1}) \\ X_{i_1} \cos (3X_{i_2}) \end{bmatrix} \end{aligned}$$

and

74 $$\begin{aligned} g(X_i)=\begin{bmatrix} 1 &\quad 0\\ 0 &\quad 1 \end{bmatrix} \end{aligned}$$

and

75 $$\begin{aligned} U_i=\begin{bmatrix} U_{i_1}\\ U_{i_2} \end{bmatrix} \end{aligned}$$

The values of the parameters used in this simulation study are given as follows.

$$\begin{aligned}K_{d_i}=\begin{bmatrix} 12 &\quad 0\\ 0 &\quad 10 \end{bmatrix}, \quad K_{a_i}=\begin{bmatrix} 10 &\quad 0\\ 0 &\quad 10 \end{bmatrix} \end{aligned}$$

The learning rate $\gamma _i=30$. We have selected RBF NN basis functions given by $\Phi (X_i)=[\phi _1(X_i) \ \phi _2(X_i)\ \ldots \ \phi _{30}(X_i)]^T$, where, $\phi _j(X_i)=exp^{-\frac{(X_i-\mu _j)^T(X_i-\mu _j)}{\psi _j^2}}$. The centers of the basis functions are spaced evenly in the range of $[-10,10]\times [-10,10]$. The width of each basis function is selected as $\psi _j=2$. The value of $\sigma _i$ is chosen as 0.12. The disturbance added is given by

$$\begin{aligned} D_i=\left[ 20 \cos \left( \frac{\pi X_{i_1}}{2}\right) \ 0\right] ^T \end{aligned}$$

which is unknown to the controller. The state trajectories of all the agents are shown as $X_1$ and $X_2$, where, $X_1=[X_{1_1}\ X_{2_1}\ \ldots \ X_{10_1}]$ and $X_2=[X_{1_2}\ X_{2_2}\ \ldots \ X_{10_2}]$. Similarly, the controls for the agents are shown by $U_1=[U_{1_1}\ U_{2_1}\ldots \ U_{10_1}]$, and $U_2=[U_{1_2}\ U_{2_2}\ldots \ U_{10_2}]$. Also, the virtual states are given by $X_{a_1}=[X_{a1_1}\ X_{a2_1}\ldots \ X_{a10_1}]$, and $X_{a_2}=[X_{a1_2}\ X_{a2_2}\ldots \ X_{a10_2}]$. The initial values of the states of all the agents are given in Table 1.

**Table 1:** Initial conditions of the states of the agents.

$X_1$	2	− 2	− 2	− 1	9	3	− 1	6	5	− 5
$X_2$	0	− 1	4	1	0	− 5	7	8	− 3	4

The adjacency matrix is given by

$$\begin{aligned}A=\begin{bmatrix} 0 &{}0 &{}1 &{}1 &{}1 &{}1 &{}0 &{}1 &{}1 &{}1 \\ 0 &{}0 &{}0 &{}0 &{}0 &{}0 &{}0 &{}1 &{}0 &{}1 \\ 1 &{}0 &{}0 &{}0 &{}0 &{}0 &{}1 &{}0 &{}0 &{}1 \\ 1 &{}0 &{}0 &{}0 &{}1 &{}1 &{}0 &{}0 &{}0 &{}1 \\ 1 &{}0 &{}0 &{}1 &{}0 &{}1 &{}0 &{}1 &{}0 &{}0 \\ 1 &{}0 &{}0 &{}1 &{}1 &{}0 &{}1 &{}1 &{}1 &{}0 \\ 0 &{}0 &{}1 &{}0 &{}0 &{}1 &{}0 &{}1 &{}1 &{}0 \\ 1 &{}1 &{}0 &{}0 &{}1 &{}1 &{}1 &{}0 &{}0 &{}1 \\ 1 &{}0 &{}0 &{}0 &{}0 &{}1 &{}1 &{}0 &{}0 &{}1 \\ 1 &{}1 &{}1 &{}1 &{}0 &{}0 &{}0 &{}1 &{}1 &{}0 \end{bmatrix}\end{aligned}$$

**Figure 4:**
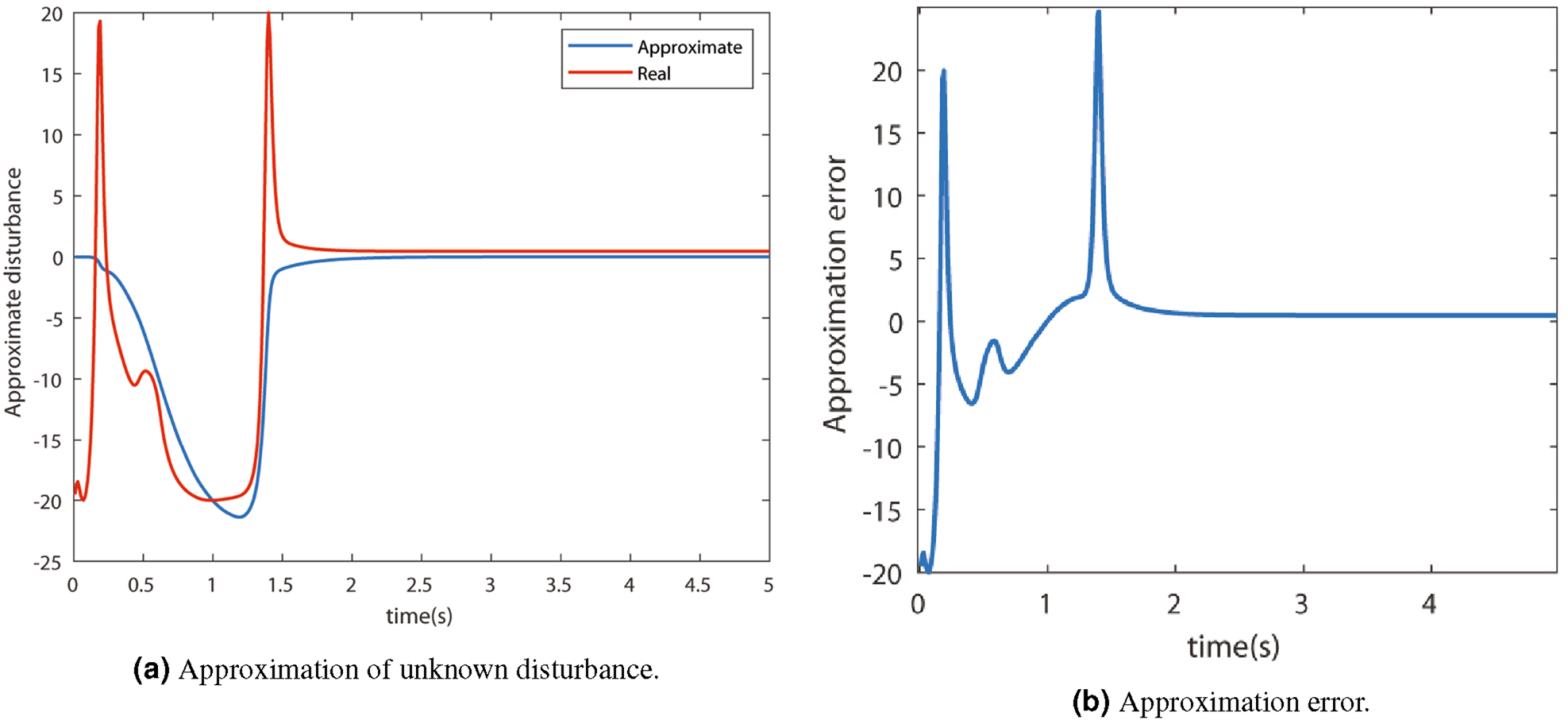
Performance of N-DNDI in approximating unknown external disturbance.

The unknown external disturbance is approximated by a neuro-adaptive controller. The approximated and real disturbance is shown in Fig. 4a and the approximation error is shown in Fig. 4b.

**Figure 5:**
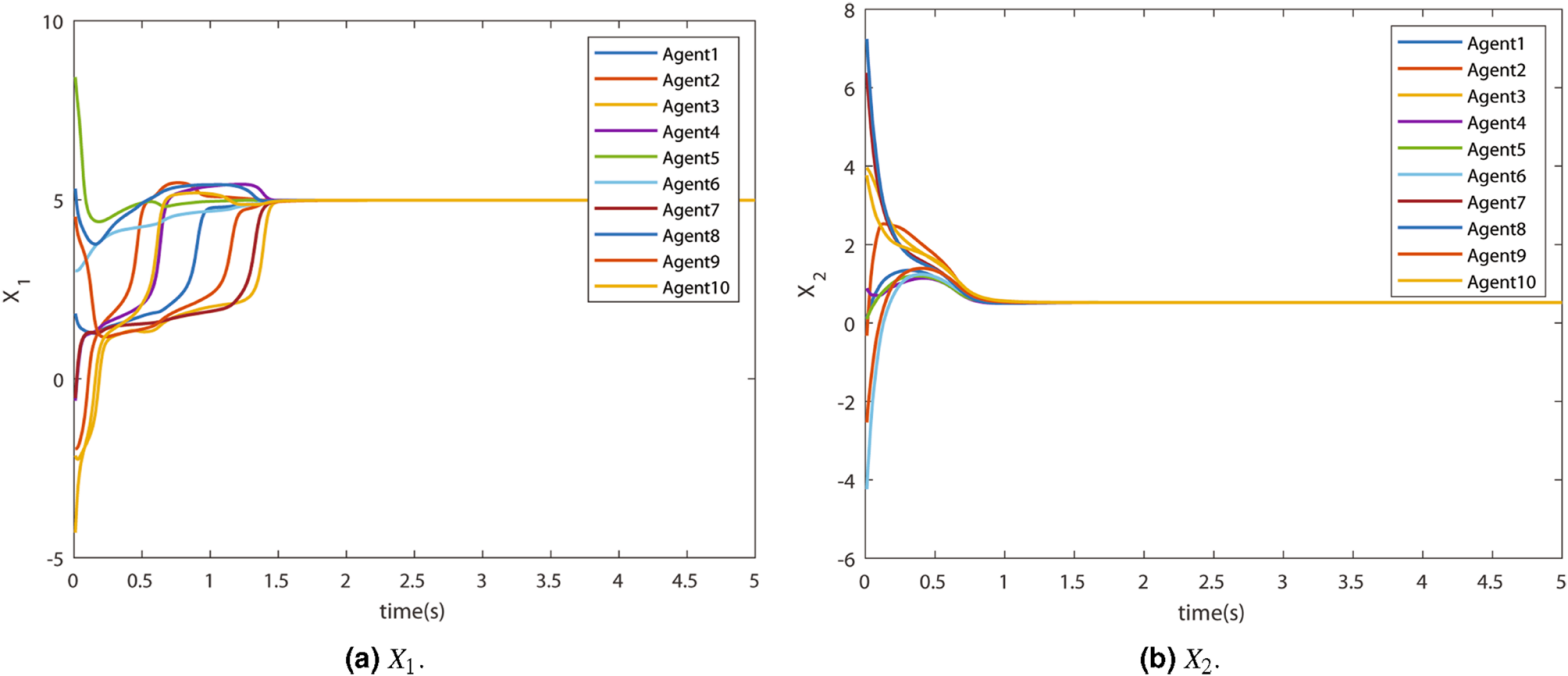
Actual state trajectories.

It can be observed that the approximation is very good, which can be confirmed using the approximation error plot. Consequently, the states of the agents achieved the consensus in a few seconds. The state trajectories of all the agents, i.e., $X_{1}$ and $X_2$, are shown in Fig. 5a and 5b respectively. The states of the agents reach the consensus in finite time.

**Figure 6:**
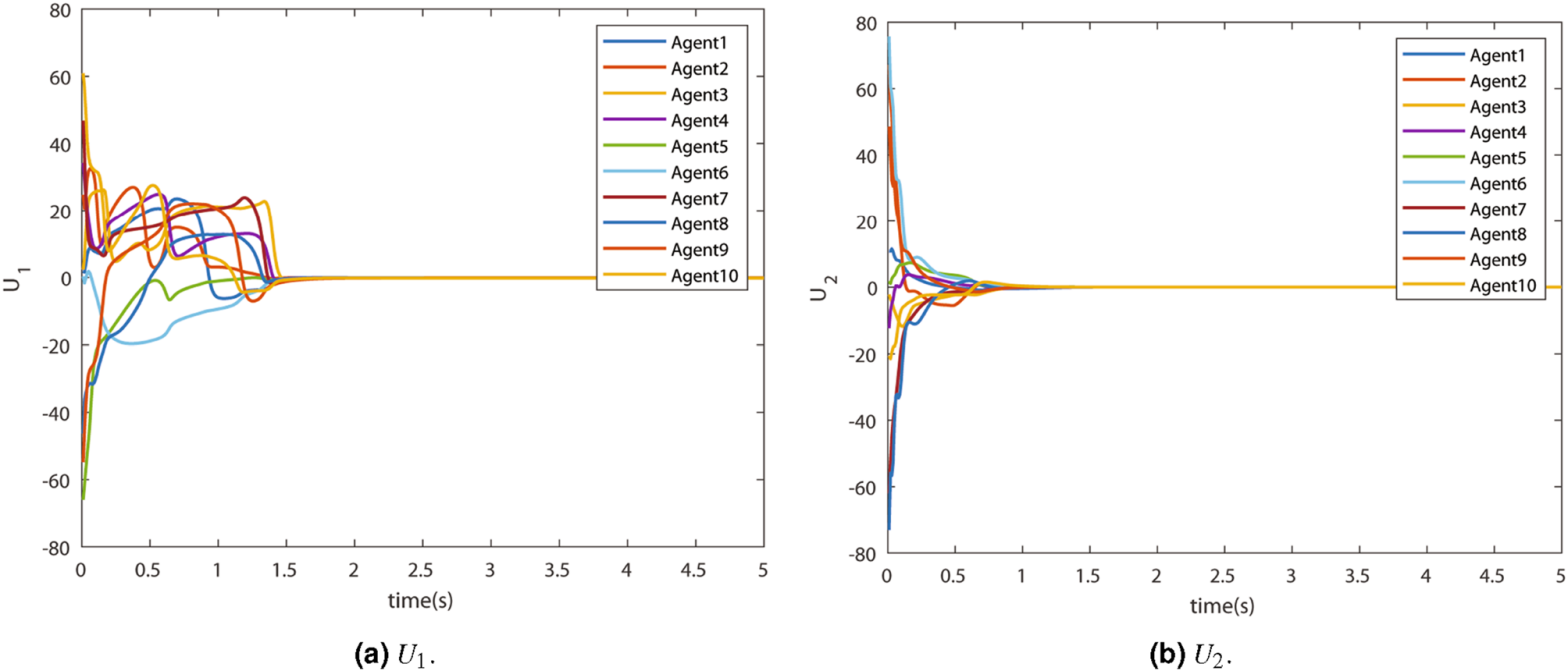
Neuro-adaptive control.

The consensus is achieved by neuro-adaptive consensus controls $U_1$ and $U_2$ which are shown in Fig. 6a and 6b respectively.

**Figure 7:**
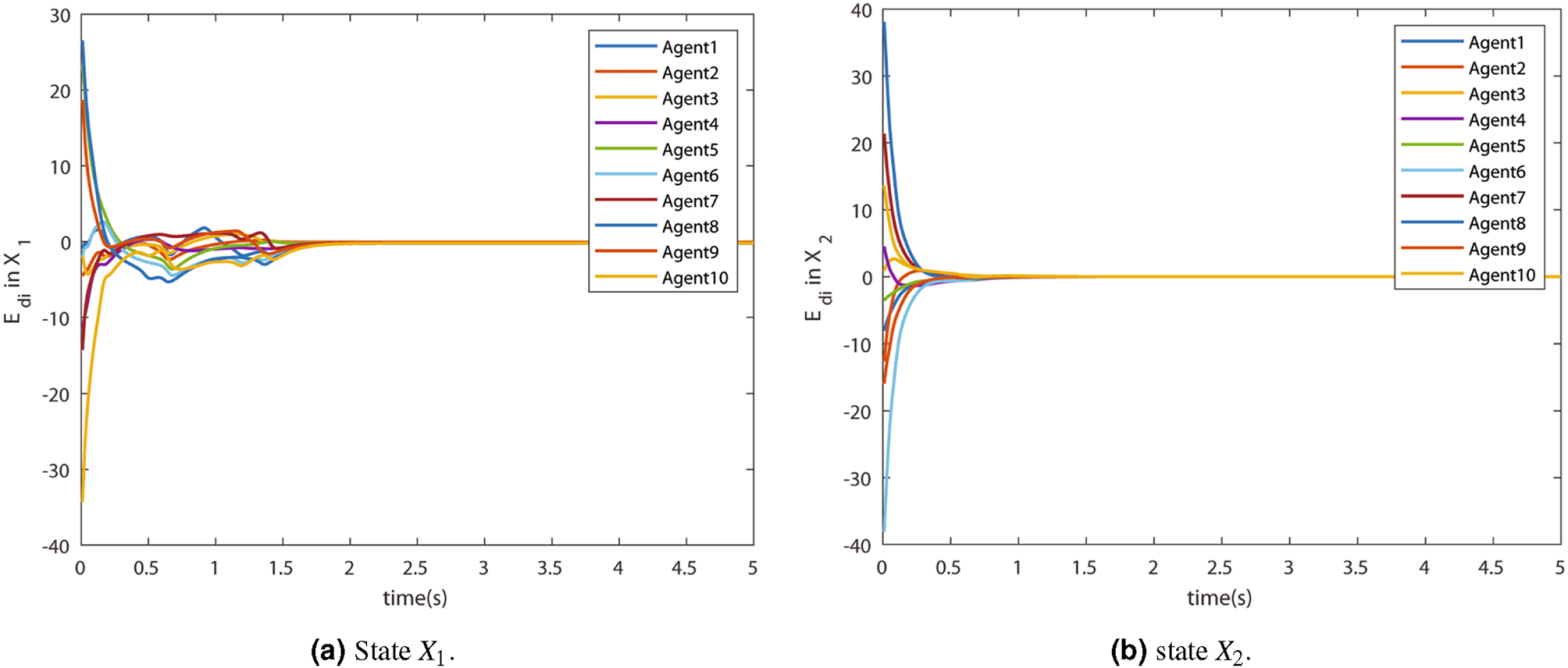
Consensus error $$E_{di}$$.

The convergence of the states is shown by the consensus errors $E_{di}$ in state $X_1$ and $X_2$. They are shown in Fig. 7a and 7b respectively. The errors converged in a few seconds. This means the virtual states $X_{a1}$ and $X_{a2}$ successfully reach the consensus.

**Figure 8:**
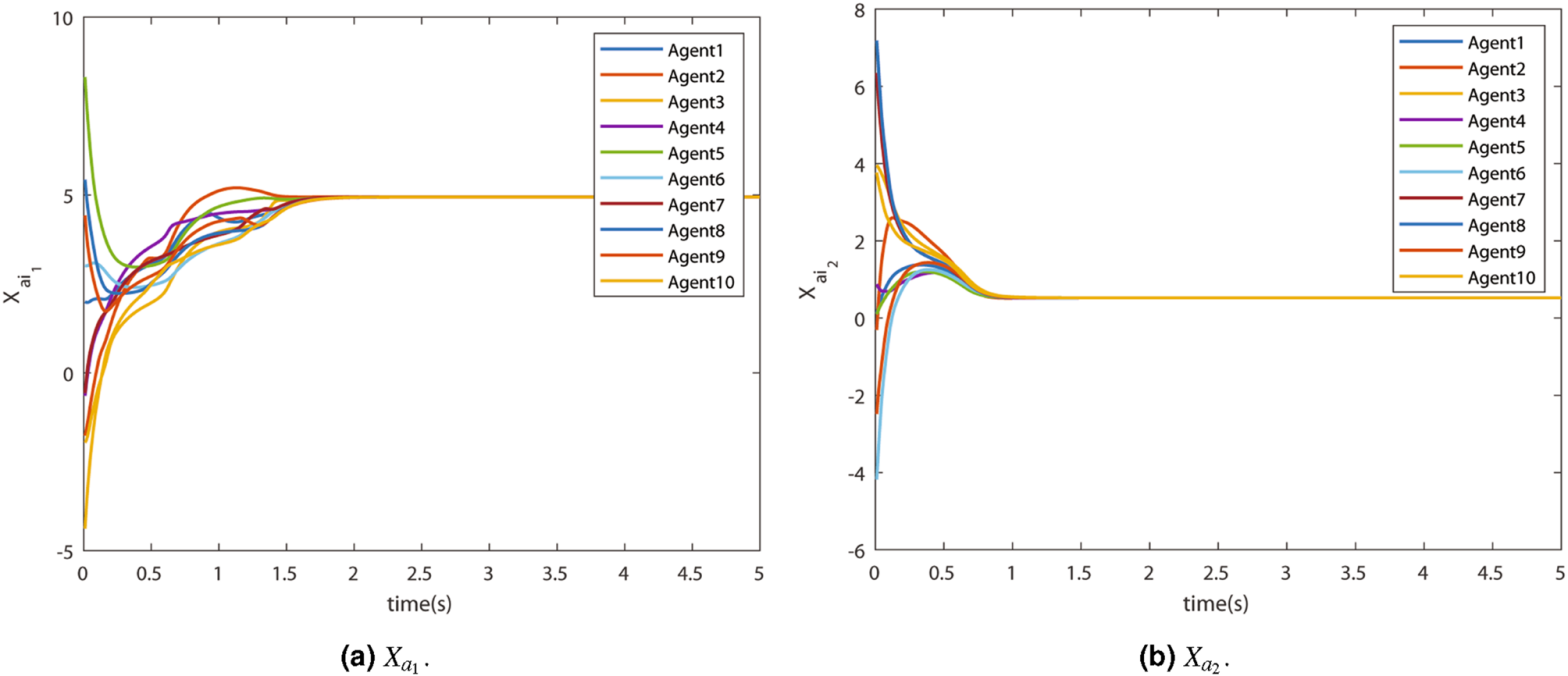
Virtual state trajectory.

The virtual states $X_{a_1}$ and $X_{a_2}$ are shown in Fig. 8a and 8b respectively. It can be observed that the consensus value of the virtual state and the actual states are the same. Therefore, the actual states tracked the virtual states accurately. The proof of the tracking can be given by virtual errors.

**Figure 9:**
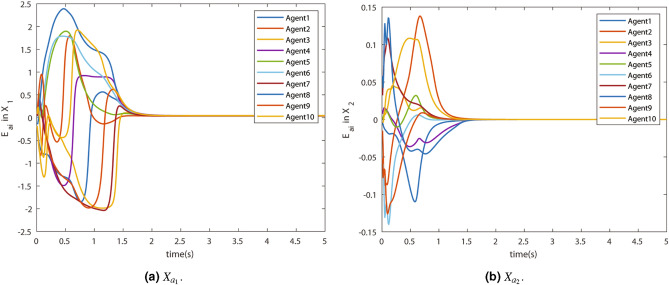
Virtual error $$E_{ai}$$.

The virtual errors $E_{ai}$ in states $X_1$ and $X_2$ are shown in Fig. 9a and 9b respectively. They have converged in finite time.

## Conclusion

The augmentation of neuro-adaptive structure to distributed nonlinear dynamic inversion (DNDI) frame produces a unique adaptive controller (N-DNDI) that efficiently handles the external disturbance. The N-DNDI inherits the features of the NDI technique and handles the unknown external disturbance. The convergence study provided in this paper explains the correctness of the design. The simulation results show that the neural network embedded in the controller approximates the unknown external function and the DNDI controller computes the consensus control signal accordingly. Consequently, the consensus is achieved in finite time. Hence, the proposed N-DNDI is a deserving candidate for consensus control in the presence of unknown external disturbances. We consider the heterogeneous agents along with communication issues as part of our future research plan. Also, we will present a comparison study of the proposed controller with the existing controllers.
